# Acupuncture modulates the AMPK/PGC-1 signaling pathway to facilitate mitochondrial biogenesis and neural recovery in ischemic stroke rats

**DOI:** 10.3389/fnmol.2024.1388759

**Published:** 2024-05-15

**Authors:** Kaixin Guo, Yan Lu

**Affiliations:** Department of Acupuncture, Shandong University of Traditional Chinese Medicine, Jinan, China

**Keywords:** ischemic stroke, acupuncture, AMPK/PGC-1α, mitochondrial energy metabolism, oxidative stress

## Abstract

**Aims:**

The main objective of this study was to investigate the role and mechanism of acupuncture on anti-nerve injury in the acute phase by regulating mitochondrial energy metabolism via monophosphate-activated protein kinase (AMPK)/peroxisome proliferator-activated receptor-γ coactivator 1α (PGC-1α) axis in rat ischemic stroke.

**Main methods:**

Middle cerebral artery occlusion (MCAO) was established by middle cerebral artery occlusion/reperfusion. One-week of acupuncture was performed during the acute phase of ischemic stroke. The neurological function and brain tissue integrity were evaluated. Mitochondrial function (intracellular ATP level and the activity of mitochondrial respiratory chain complex I) and the level of NADH oxidase (NOX) were detected by enzymatic chemistry. Next, the potential molecular mechanisms were explored by western blotting, fluorescence quantitative PCR and immunohistochemistry method.

**Key findings:**

(1) Acupuncture treatment for MCAO/R rats showed a significant improvement in the infarcted tissue accompanied by functional recovery in Zea-Longa score and balance beam score outcomes, motor function performances. (2) Acupuncture increased the levels of ATP and mitochondrial respiratory chain complex I, decreased the NOX levels in cerebral ischemia established by suture-occluded method. (3) Acupuncture reduced the necrosis dissolution of neuronal cells and meningeal edema, while promoting angiogenesis. (4) Quantitative immunohistochemical staining results showed acupuncture can increase the expression of AMPK, p-AMPK and the mitochondrial transcription factor PGC-1α, NRF2, TFAM and uncoupling protein 2 (UCP2). Meanwhile, acupuncture treatment up-regulated the expression of the corresponding protein. (5) Subsequently, acupuncture enhanced AMPK phosphorylation as well as the expression of PGC-1α, NRF2, TFAM and UCP2, implicated in mitochondrial synthesis and cellular apoptosis. (6) Finally, injections of AMPK antagonists and activators confirmed AMPK as a therapeutic target for the anti-nerve damage effects of acupuncture.

**Significance:**

Acupuncture intervention relieved ischemic stroke progression in MCAO rats by promoting energy metabolism and mitochondrial biogenesis in the brain and alleviating neuronal apoptosis, which was mediated by eliciting AMPK/PGC-1α axis, among them AMPK is a therapeutic target.

## 1 Introduction

Stroke has become a major challenge in global medicine due to its high morbidity, high mortality rate, high disability rate and high recurrence rate. More than 15 million people are affected worldwide every year, bringing great health and economic pressure to individuals, families and society. It is reported that ischemic stroke accounts for 71% of all stroke cases (Feigin et al., 2018), while the prevalence in Americans is ~85%−87% (Benjamin et al., 2019). Symptoms such as dizziness, nausea and hemianesthesia due to mild ischemia can be relieved; in severe cases, it causes hemiplegia and cognitive dysfunction, even irreversible damage such as death. Hemiplegia after stroke is one of the primary functional disorders in patients with stroke. It is also a key factor in various poor clinical prognosis. The causes of brain injury include neuroinflammation, oxidative stress and mitochondrial dysfunction (Jiang et al., 2018; Tang et al., 2020) and this paper mainly focuses on the latter two. Any form of “energy deprivation” of the central nervous system, such as ischemia, trauma, and infection, is closely associated with energy metabolism. Mitochondria are the center of cellular energy metabolism, and the maintaining normal function of mitochondria is important for cell energy homeostasis and normal activity of organisms (DeBalsi et al., 2017; Baron, 2018; Popov, 2020). It is well known that cerebral ischemia/hypoxia is accompanied by changes in ATP and NOX levels, meanwhile the changes in ATP and ROS-induced energy stress strongly activates monophosphate-activated protein kinase (AMPK) (Wu and Zou, 2020). AMPK has been demonstrated to attenuate oxidative stress, apoptosis and inflammation (Toyama et al., 2016). As a cellular energy sensor, AMPK is also an upstream regulator of PGC-1 α, plays a role in promoting energy decomposition and inhibiting energy synthesis after ischemic stroke, thereby alleviating cerebral neurological damage and promoting the recovery of related motor functions. Peroxisome proliferator-activated receptor-γ coactivator 1 α (PGC-1 α) is a powerful transcriptional regulator of genes in mitochondria, activates a variety of nuclear receptors and is involved in the activation of numerous transcription factors (Suwa et al., 2008; Tsai et al., 2015), regulates processes such as mitochondrial biogenesis, cellular glycolipid metabolism, apoptosis, and oxidative stress. Their overexpression can compensate for the loss of mitochondria in neurodegenerative diseases, and can upregulate the expression of antioxidant enzyme classes, scavenge free radicals, improve the antioxidant stress capacity of rats, and reduce the degenerative death of nerve cells (Wareski et al., 2009; Ye et al., 2016). Our laboratory found that acupuncture induced high expression of AMPK in the periphery of cerebral infarct lesions in middle cerebral artery occlusion (MCAO) rats, therefore, reasonable to hypothesize that AMPK/PGC-1α axis is an important pathway in the recovery of stroke. Hence, the relationship between this pathway and mitochondrial energy metabolism can help to further explore the pathogenesis and progression of ischemic stroke.

Acupuncture, a medical technique practiced for more than 3000 years in Asian countries, was recognized by the World Health Organization (WHO) in 2003 as an effective treatment for multiple diseases (Zhu et al., 2019). Currently, the WHO approved acupuncture for improving conditions such as low back pain, migraine, osteoarthritis, depression, and stroke (Shen et al., 2016; Xing et al., 2018). Multiple clinical trial data and meta-analyses have shown that acupuncture improves CNS injury and other clinical outcomes, including stroke (Hung et al., 2019), Alzheimer's disease, Parkinson's disease (Shin et al., 2017), and spinal cord injury (Xiong et al., 2019). Traditional Chinese medicine theory holds that Fengfu (GV16, it is located 1 cun above the median of the posterior hairline, in the depression of the lower side of the external occipital carina), Baihui (GV20, it is located at the intersection of the midline on the top of the head and the line connecting the tips of the two ears) and bilateral Fengchi (GB20, it is located inferior to the occiput, in the depression between the sternocleidomastoid and the upper end of the trapezius) were first recorded in the ancient book “Taiping Shenghui Fang,” which combined with clinical empirical findings, together with Zusanli (ST36, it is located in the lower four transverse fingers of the lateral knee and the edge of the tibia) and Quchi (LI11, It is located at the midpoint of the line between LU5 and the lateral epicondyle of the humerus) can effectively promote the recovery of neuromotor and conscious functions in stroke patients. There are reports that acupuncture can improve neurological scores in stroke patients (Zhu et al., 2019; Yu et al., 2021). Multiple experimental studies also showed that acupuncture improved neurological function, muscle strength recovery, tissue perfusion, alleviated brain edema, and preserved blood-brain barrier integrity in patients with ischemic stroke (Liu et al., 2009; Yan and Hui-Chan, 2009; Li et al., 2019). Despite these clear clinical effects, the molecular mechanism by which acupuncture promotes neurological recovery in patients with ischemic stroke has not been elucidated. In the present study, we stabbed experimental animals after ischemic brain injury to determine their effects on mitochondrial function and energy metabolism levels and AMPK/PGC-1α signal pathway in the brain neural network.

This study aims to evaluate the role of acupuncture in the neural tissue injury induced by MCAO. We hypothesized that acupuncture plays an important role in regulating mitochondrial function and the level of energy metabolism after stroke through the AMPK/PGC-1α signal pathway, thereby participating in the recovery and development of neurological damage after stroke, and AMPK is a critical target for treatment. To provide key insights into the clinical application of acupuncture to promote the recovery of nerve injury in stroke patients.

## 2 Materials and methods

### 2.1 Experimental animals

Animals: Following the Laboratory Animal Guide (GB/T358922018), the study protocol was approved (License Number: Traditional Chinese Medicine-20200287). Adult SD (Sprague Dawley) male rats (230–270 g) were purchased from the laboratory animal center of Beijing huafukang Laboratory Animal Technology Co., Ltd (Beijing, China), license number: scxk (Beijing) 2020-0004. All animals were fed in cages with free access to water, naturally illuminated according to circadian rhythm, room temperature 20–26°C, and relative humidity 50%−70%.

### 2.2 Middle cerebral artery occlusion

**Figure 1:**
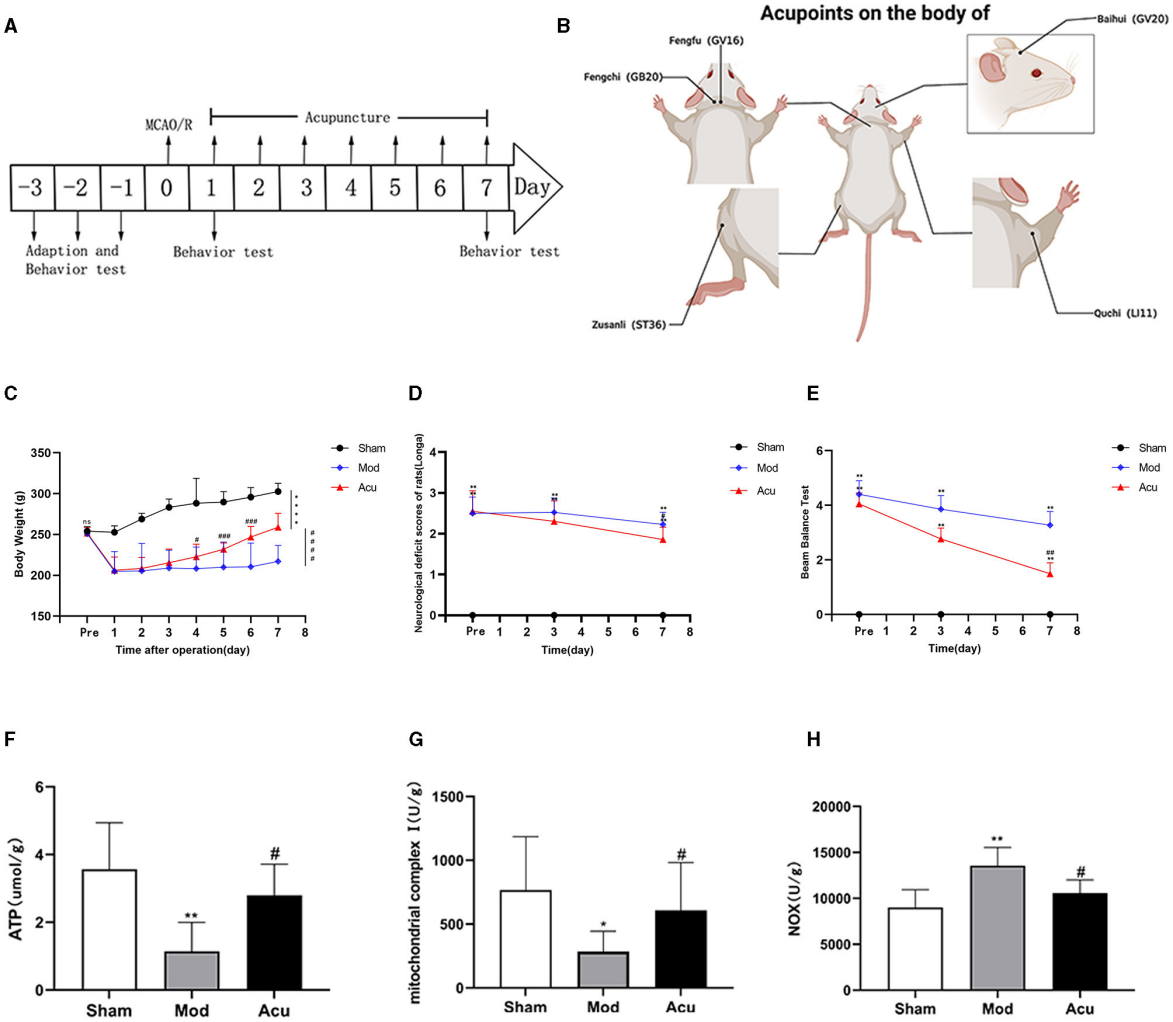
Acupuncture can improve the weight and neurological recovery of rats. **(A)** Schematic representation of the experimental timeline. **(B)** Location of acupuncture points in rats. **(C)** The weight changes of rats in each group at 7 days and the comparison after treatment (*n* = 10, one-way ANOVA). **(D, E)** Neurological scores of rats from each group on days 1 and 7, **(D)** Longa **(E)** Beam Balance Test (*n* = 10, one-way ANOVA). **(F–H)** Effect of acupuncture intervention on energy metabolism in the brain of MCAO rodents **(F)** ATP, **(G)** Mitochondrial complex I, **(H)** NOX (*n* = 10, one-way ANOVA). All data are presented as the means ± SEM; *P < 0.05, **P < 0.01, ****P < 0.0001 vs. Sham; ^#^*P* < 0.05, ^##^*P* < 0.01, ^###^*P* < 0.001, ^####^*P* < 0.0001 vs. Mod.

After 3 days of adaptive feeding of experimental animals, the MCAO model was established. Rats were anesthetized with 5% pentobarbital sodium (100 mg/kg, ip) and immobilized on a surgical platform in a supine position. A midline longitudinal incision was made in the neck to expose the right common carotid artery, after ligation of the proximal end of the common carotid artery by thread, a small port was cut at ~3 mm from the bifurcation of the internal and external carotid arteries, and a filament plug was inserted (the filament tip diameter 0.2 m and length 5 cm, model number: msrc37b200p50, source: Shenzhen RuiwADE Life Technology Co., Ltd, China). A slight resistance was encountered by pushing the filament plug into the internal carotid artery 19–21 mm along the scissor, indicating that the head had reached the proximal wall of the anterior cerebral artery, which was the origin of the middle cerebral artery, and the filament was ligated at the tethering plug. The occlusion line was pulled out after 1 h of ischemia to achieve reperfusion, the tissue was restored *in situ*, the incision was closed using full-thickness sutures, and the rats was treated with anti-infection treatment postoperatively. The sham group did the same procedure, but no filament plugs were inserted. Zea-Longa score was used for the evaluation, with scores of 1–4 classified as valid MCAO model. The animal groupings are shown in Figure 1A.

### 2.3 The experimental groups are as follows: (*n* = 10 in each group)

(1) Sham group: only isolated the carotid artery, not the middle cerebral artery thrombolysis.
(2) Mod group: Middle cerebral artery was embolized for 1 h with reperfusion.
(3) Acupuncture group: acupuncture was done for 7 days after mold making.

### 2.4 Acupuncture stimulation

Acupuncture is performed by an acupuncturist, following the National GB/T21709.4-2008 Technical Operating Standard of Acupuncture, Part 4. Fengfu (GV16), Baihui (GV20), bilateral Fengchi (GB20), Zusanli (ST36), and Quchi (LI11) were selected. GV16 locates at occipital posterior occipital ridge atlas joint. GV20 locates in the middle of the parietal bone. GB20 locates at the inferior occipital side about 3 mm from the posterior midline. ST36 locates posterolateral to the knee joint, ~3 mm below the capitulum fibula. LI11 locates in a depression in the lateral anterior aspect of the joint of the proximal radius (Figure 1B). In the acupuncture group, to ensure smooth acupuncture, to restrain the rats with a soft cloth, Baihui point was inserted into the needle at 2 mm depth, while and the other acupoints are inserted vertically at 3 mm depth and fixed for 20 min. Acupuncture was started from the day after mold making and treated once a day for 1 week. All mice in the other groups maintained the same grasping and fixation.

### 2.5 Neurological deficit score

(1) Neurological deficit was scored 24 h after the animals were awake and after the completion of acupuncture treatment by reference to the neurological score method of Zea longa (Longa et al., 1989; Park et al., 2021). The criteria are as follows: 0 points: no neurological defect symptoms; 1 point: the right forelimb of the rat cannot be fully extended; 2 points: the right circle when walking freely; 3 points: the rat falls to the right when walking freely; 4 points: the rat cannot walk spontaneously, or loss of consciousness.
(2) Behavioral testing of the rats was performed using the balance beam test with an adaptive training session of 20 min per day for three consecutive days before the modeling surgery, the purpose is to let rats adapt to the test environment and behavioral test content, to minimize the stress response in the behavioral test, improve compliance, make the experimental results more reliable.

The balance beam experiment was performed in each group 24 h and after acupuncture treatment. It was mainly used to evaluate the coordinated motor function of the rodent limbs (Takata et al., 2006). The apparatus used consisted of a square horizontal wood (2.5 cm × 122 cm × 42 cm) connected to a black box (20.5 cm × 25 cm × 25 cm). A strong beam of light placed above the starting point prompted the rat to walk across the horizontal log. Each rat should be trained three times before the formal experiment, so that each rat can pass the balance beam normally, including rats that cannot pass the balance beam within 1 min. The scoring standard is as follows: 0 point: through the balance beam, not falling; 1 point: through the balance beam, the hind limb fall rate is <50%; 2 points: through the balance beam, but the hind limb drop rate is more than or equal to 50%; 3 points: can cross the balance beam, but the opposite side of the lesion cannot help to move forward; 4 points: sit on the horizontal beam, but cannot crawl; 5 points: cannot stay on the horizontal wood, fall. Each rat was tested three times daily at a fixed time, and the average of the three scores was used for statistical analysis, and those without coordinated motor dysfunction of the limbs were removed. After neurological function score, animals were killed by excessive anesthesia.

### 2.6 Histopathological analysis of brain

The brain tissues in the three groups were quickly removed, immediately frozen in liquid nitrogen, and then transferred to an −80°C refrigerator until used for further experiments. Among them the animal's brain tissues (*n* = 6) were quickly fixed in 4% paraformaldehyde fixative solution in good condition. Then trimmed strictly according to the normative procedure for pathological experimental testing. Next, decalcification, dehydration, transparency, wax immersion, tissue embedding, and sectioning (section thickness 4 μm) were performed. After deparaffinization in xylene and rehydration in graded ethanol, sections were sequentially stained with hematoxylin and eosin and evaluated with an Eclipse ci-l upright light microscope (Nikon Tokyo, Japan).

### 2.7 ELISA assay to detect the cytokines level

We examined cytokine levels in each group of rats (*n* = 10). The levels of cytokines in brain tissues and supernatant cells were detected following the instructions of ELISA kits [ATP (BC0305), Mitochondrial complex I (BC0515), NOX (BC0635), Beijing Solarbio Science & Technology Co., Ltd, Beijing, China].

### 2.8 Immunostaining and image analysis

Paraffin embedded brain tissues (*n* = 6) was cut into sections of ~5 μm thickness using a pathology microtome (Shanghai Leica Instruments Co., Ltd.). They were deparaffinized, rinsed in distilled water, antigen retrieval, rinsed three times in PBS and incubated in blocking solution 5% BSA for 20 min, sections were incubated with AMPK β 1 (Abclonal, A12491, 1:100), P-AMPK β 1 (Abclonal, AP1195, 1:100), TFAM (Abclonal, A13552, 1:1 00), PGC1 α (Abclonal, A12348, 1:100), NRF2 (Abclonal, A0674, 1:100), UCP2 (Servicebio, GB11377, 1:100) in a wet box at 4°C overnight. Next, it was rewarmed for 30 min, rinsed three times in PBS, secondary antibodies were added dropwise and incubated for 1 h at room temperature. Then rinsed three times in PBS, developed with freshly configured DAB, counter-stained with hematoxylin, and mounted with dehydrated, transparent, and neutral gum. Finally, five fields of positive expression were randomly taken from each section at 400 × magnification, and the software Image J (National Institutes of health, USA) was used to measure and calculate the average absorbance value.

### 2.9 PCR reverse transcription-quantitative polymerase chain reaction

Three appropriate amounts of tissue were taken from each group, and total RNA was extracted using RNA extraction solution (Servicebio, Wuhan, China) according to the manufacturer's instructions, and the RNA concentration was detected with Nanodrop 2,000 (Thermos); a bio reverse transcription kit (Servicebio, Wuhan, China) was used, and a reaction system was set up for RNA reverse transcription to synthesize cRNA. Subsequently, polymerase chain reaction (PCR) amplification was performed with the following amplification conditions: pre denaturation at 95°C for 10 min; 95°C for 15 s, 60°C for 60 s, 40 cycles; 75–95°C with 1°C ramp every 20 s. GAPDH was used as internal reference and the primers were obtained from Wuhan Google Biotechnology Co, Ltd, and the primer sequences are shown in Supplementary Table 1. Relative gene expression was calculated using the double standard curve method. Three independent experiments were repeated.

### 2.10 Western blot analysis

Total protein was extracted from brain tissue collected from the ischemic cortical region of rats using the conventional total protein extraction method. Rat brain tissues (*n* = 4), which had been preserved before retrieval, were extracted with lysis solution (Servicebio, Wuhan, China) for total protein, and the protein concentration was determined by the BCA protein quantitative assay kit (Servicebio, Wuhan, China). Samples were denatured in boiling water, and proteins were separated by electrophoresis and transferred to a methanol activated PVDF membrane (0.45 μm). Nonfat milk was added and blocked for 30 min at room temperature; with antibody to AMPK β 1 (Abclonal, A12491, 1:1,000), P-AMPK β 1 (Abclonal, A12491, 1:1,000), TFAM (Abclonal, A13552, 1:1,000), PGC1 α (Abclonal, A12348, 1:1,000), NRF2 (Abclonal, A0674, 1:1,000), UCP2 (Servicebio, GB11377, 1:1,000) were incubated overnight at 4°C, followed by the addition of secondary antibody reaction for 30 min at room temperature. The protein bands were chemiluminescent with the addition of mixed ECL solution, followed by development with developing, developing reagents, and processing with Photoshop and Alpha software to analyze the densitometric values of the target bands.

### 2.11 Inhibitors and agonists were injected into the lateral ventricles

Animals were randomly divided into the following four groups (10 rats in each group): MCAO, MCAO + Acu, MCAO + Acu + AMPK antagonist (compound C), and MCAO + Acu + AMPK agonist (AICAR). The compound C and AICAR were provided by Abclonal under catalog numbers A22182 and A5559, respectively. The molding method was the same as before. A 5 μl test solution was injected with a 10 μl Hamilton microliter syringe. The concentrations of Compound C and AICAR were 100 nmol/5 μl, and 1 nmol/10 μl respectively. A simple marker was made 1.0 mm after the anterior fontanelle and 2.0 mm beside the midline, where a 1 mm diameter hole was drilled with a dental drill. The 5 μl antagonist or agonist were absorbed into the vertical target bone hole for 3.5 mm, and the injection speed was 0.5 μl/min. After retaining the needle for 5 min, it was slowly withdrawn within 5 min. Finally, the pinhole was sealed with bone wax to clean the wound and suture it for disinfection. Acupuncture treatment was performed the day after the injection. Functional nerve injury was assessed after completion of treatment and statistically analyzed. All rats were killed after the experiment.

### 2.12 Statistical analysis

Statistical analysis was performed with the software SPSS 26.0 (Version 26, IBM, New York, USA). All experimental data were expressed as mean ± SEM (*n* = 4–10), and mapped using GraphPad (GraphPad, San Diego, CA, USA). Body weight data were analyzed by repeated measures data ANOVA with GraphPad, comparisons between multiple groups were performed using one-way ANOVA and multiple comparisons using LSD or Tamhane's T2 analyses, and *P* < 0.05 was considered as statistically significant.

## 3 Results

### 3.1 Acupuncture alleviated neurological impairment in rats with focal cerebral ischemia

First, we observed the weight changes of three groups of rats (*n* = 10), it was found that the body weight of rats in the sham group gradually increased within 7 days. Compared with the sham group, the body weight of rats in the model group decreased significantly on the first day, but it also gradually increased with the defense of time in the last 6 days. At the end of acupuncture treatment, the body weight of rats in the acupuncture group was significantly higher than that in the model group (Figure 1C).

Then, we analyzed whether acupuncture affected nerve injury recovery and improved the Zea-Longa score and balance beam score in MCAO rats. Longa neurological score test was performed on rats from each group (*n* = 10) before and after acupuncture treatment. The results are shown in Figures 1D, E. We observed that after surgery, both neurological scores in the sham group were 0, the Longa score and balance beam score were significantly higher in the other groups relative to the sham group. One week later, the Longa score and balance beam score in the simultaneous segment model group were still significantly higher than those in the sham group, and the acupuncture intervention could significantly reduce the Longa score to 1.8 and the balance beam score to 1.5. In conclusion, acupuncture significantly improved neural function in rats with focal cerebral ischemia by a specific mechanism.

### 3.2 Acupuncture alleviates brain cell damage in MCAO rats

**Figure 2:**
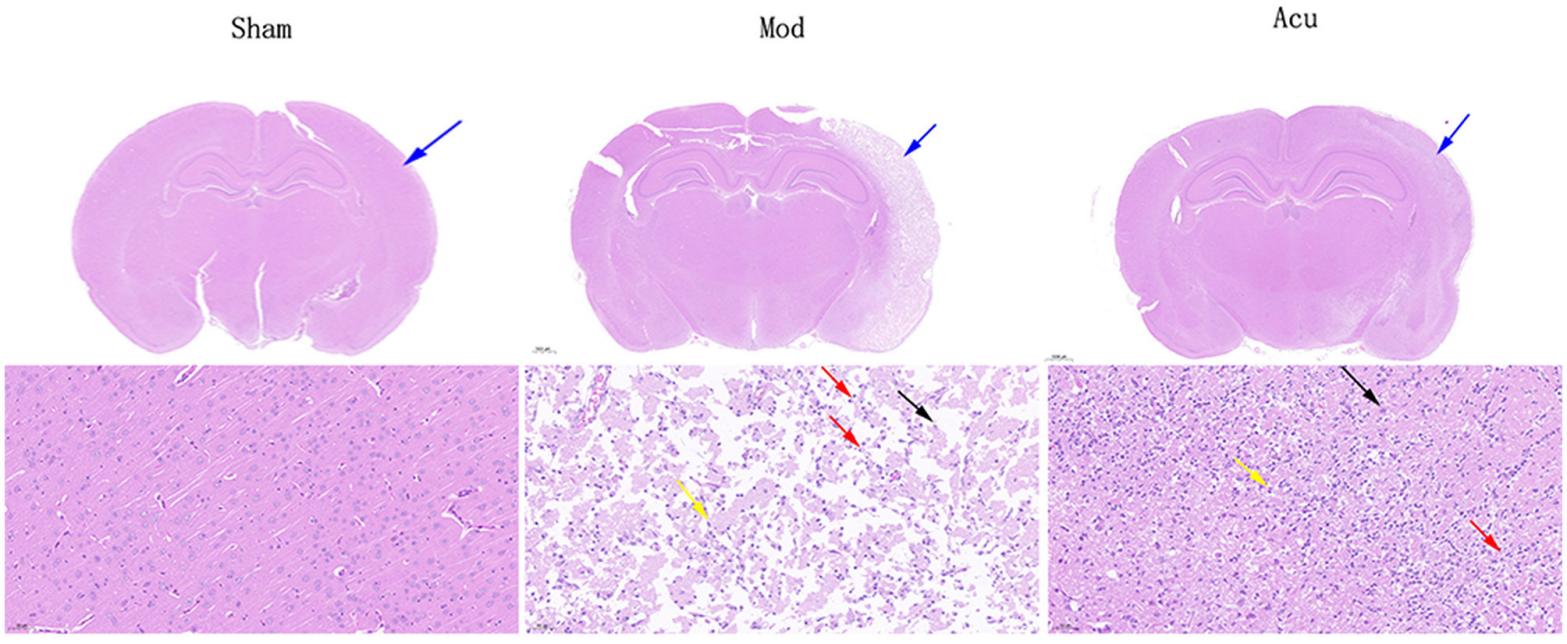
Representative HE staining of brain tissues in the three groups. Black arrow: large area of necrosis of neurons in the model group; Yellow arrow: large number of neovascularization; Red arrow: massive lymphocytes and macrophage infiltration; Blue arrow: cerebral edema and loose connective tissue.

To observe the effect of acupuncture on brain damage cells in MCAO rats, we performed HE staining of brain tissue, as shown in Figure 2. In the cerebral cortex and hippocampus of the sham group, the neurons and neurons were abundant and closely arranged, with normal morphological structure, clear cytoplasmic division and obvious nucleoli, and no obvious inflammation. In the model group, large area of necrosis of neurons (black arrow) and tissue softening, large number of neovascularization (yellow arrow), with massive lymphocytes and macrophage infiltration (red arrow), cerebral edema and loose connective tissue (blue arrow). The acupuncture group showed significant remission compared to the model group.

### 3.3 Acupuncture enhances mitochondrial function and energy metabolism levels after MCAO

Since mitochondria are the central part of cellular energy metabolism, maintaining the biological activity of mitochondria is important for energy stability in the brain after ischemia. The generation of ATP by oxidative phosphorylation is a major function of mitochondria, whereas this process mainly relies on complexes on the respiratory chain for completion. Therefore, we observed the effects of acupuncture on mitochondrial function (ATP and mitochondrial complex I) and levels of brain energy metabolism (NOX) in MCAO rats. Figures 1F–H shown that compared with the sham group, the levels of ATP and mitochondrial complex I in the model group were decreased (*P* < 0.05), however NOX was increased (*P* < 0.05). After acupuncture treatment, the ATP and mitochondrial complex I levels were significantly increased, while the NOX levels were decreased. The mitochondrial functional test results showed that acupuncture could improve the mitochondrial function and brain energy metabolism level in the brain tissue of MCAO rats.

### 3.4 Acupuncture enhanced the localized expression of factors related to the AMPK/PGC-1α pathway

**Figure 3:**
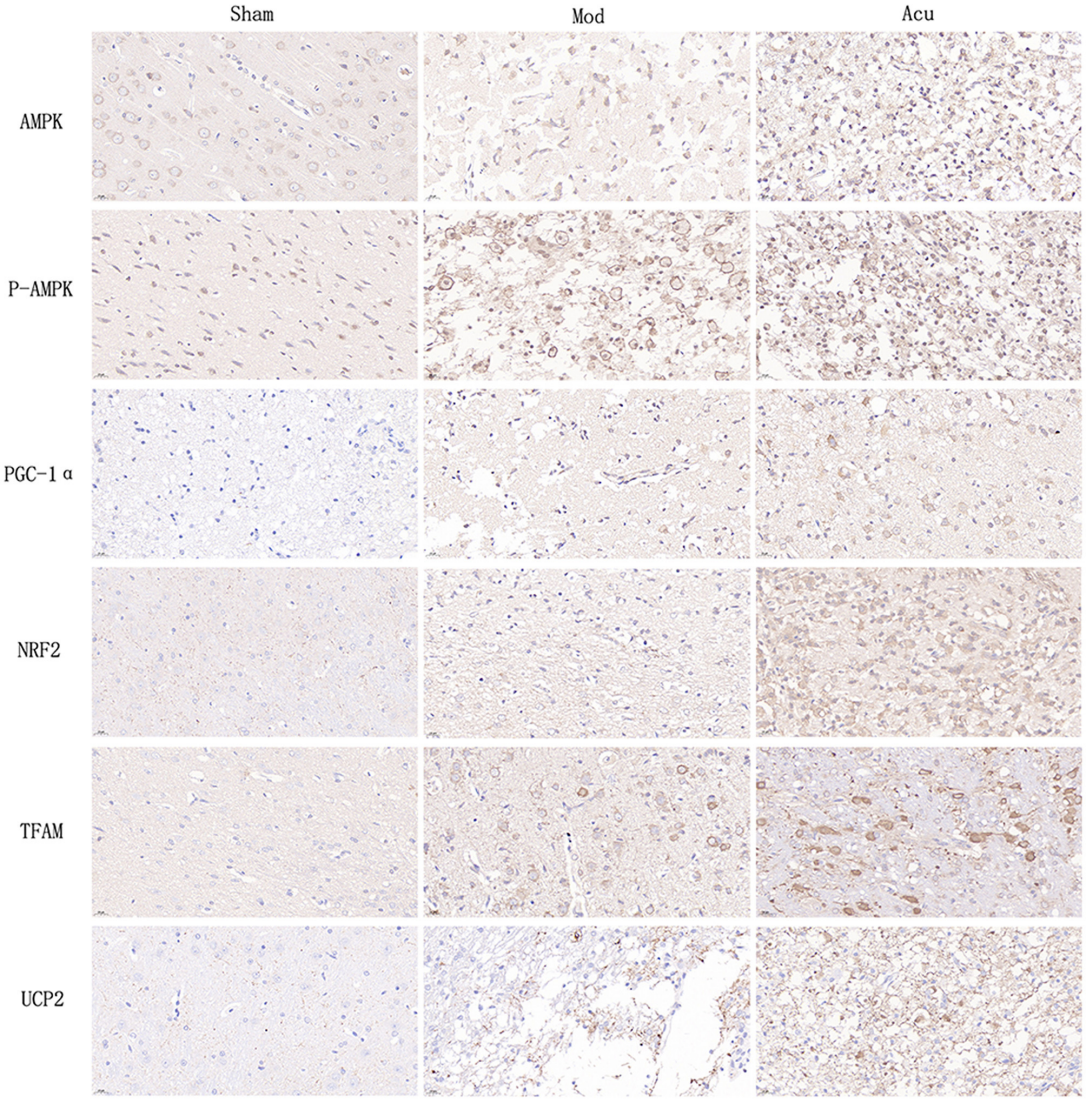
Immunohistochemical staining of various indexes in brain tissue of three groups.

**Figure 4:**
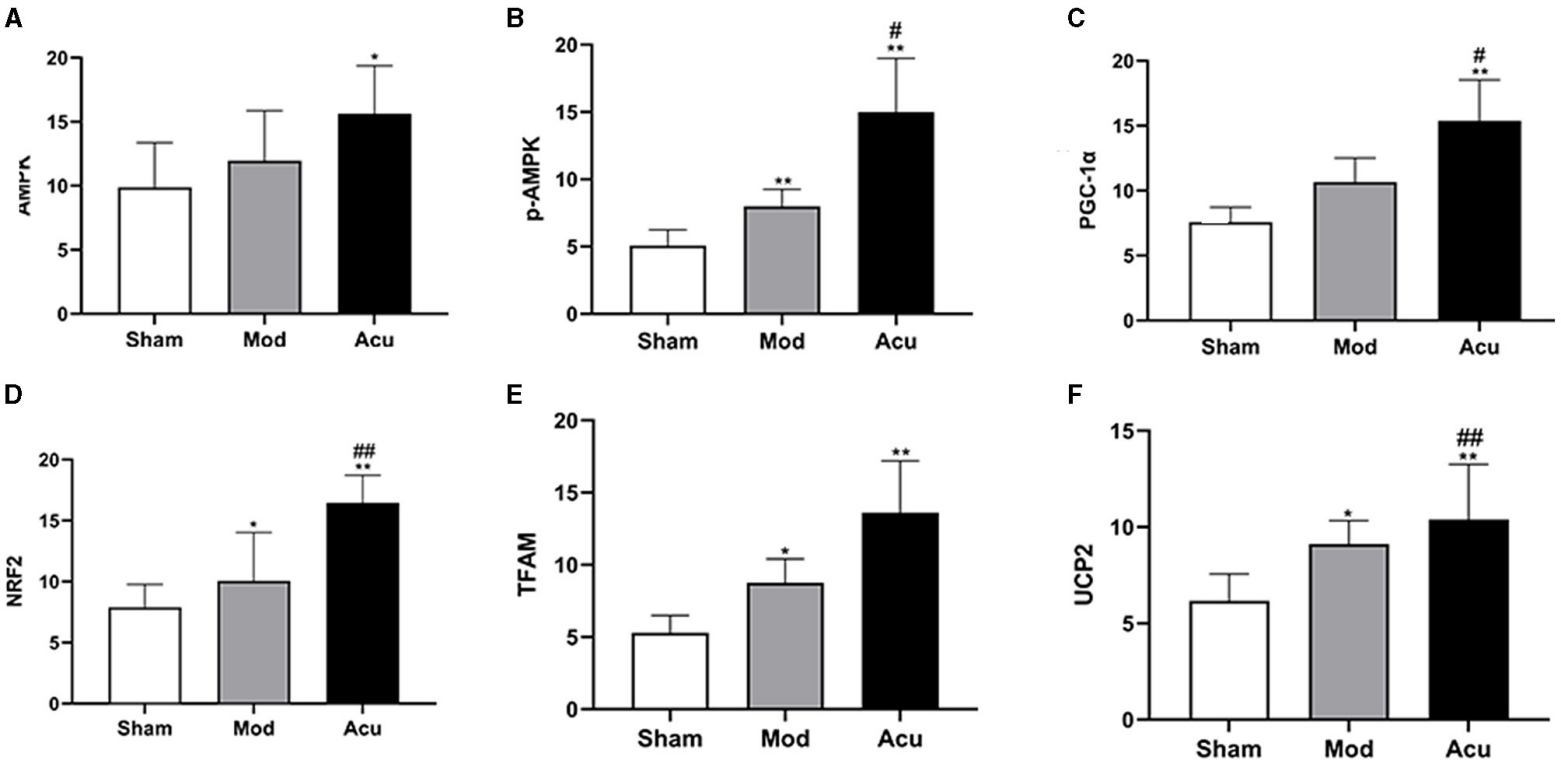
Acupuncture intervention can activate the AMPK/PGC-1α pathway in MCAO rats, **(A–F)** Average absorbance value of AMPK, p-AMPKβ1, NRF2, NRF, TFAM and UCP2 of brain tissue in the three groups (*n* = 6, one-way ANOVA). All data are presented as the means ± SEM; *P < 0.05, **P < 0.01 vs. Sham; ^#^*P* < 0.05, ^##^*P* < 0.01 vs. Mod.

Studies indicate a close relationship between ischemic stroke and the AMPK/PGC-1α pathway, as well as brain energy metabolism levels. To elucidate the molecular mechanisms underlying acupuncture's attenuation of ischemic brain injury, we conducted histological analysis to locate the expression of PGC-1α, TFAM, NRF2, UCP2, AMPKβ1, and p-AMPKβ1 in cerebral neuronal cells, particularly within the ischemic penumbra. The results are depicted in Figures 3, 4, revealing that AMPKβ1, p-AMPKβ1, PGC-1α, NRF2, TFAM, and UCP2 are predominantly expressed in the cell membrane and cytoplasm of neuronal cells. Quantitative analysis demonstrated that acupuncture significantly upregulated the expression of p-AMPKβ1, PGC-1α, NRF2, TFAM, and UCP2 in neuronal cells, whereas AMPKβ1 exhibited only a marginal increase.

### 3.5 Acupuncture increased the expression of AMPK/PGC-1α pathway related proteins

**Figure 5:**
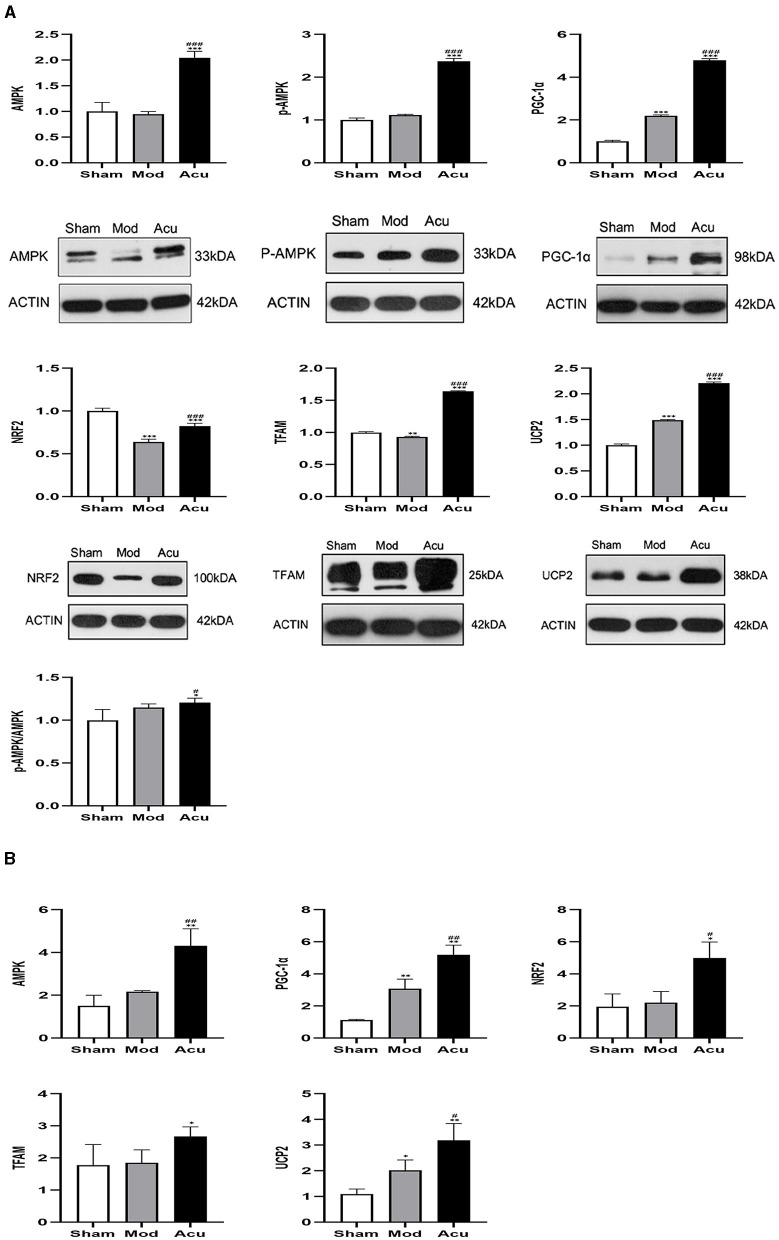
Acupuncture intervention can promote the expression of mitochondrial neogenesis related proteins and mRNA. **(A)** Expression of various proteins including AMPK, p-AMPKβ1, PGC-1α, NRF2, TFAM and UCP2 (*n* = 4, one-way ANOVA). **(B)** Expression of mRNA in AMPK, PGC-1α, NRF2, TFAM and UCP (*n* = 4, one-way ANOVA). All data are presented as the means ± SEM; *P < 0.05, **P < 0.01, ***P < 0.001 vs. Sham; ^#^*P* < 0.05, ^##^*P* < 0.01, ^###^*P* < 0.001 vs. Mod.

To observe the effect of acupuncture on the protein expression of factors related to mitochondrial biosynthesis in the brain tissue of MCAO rats, we examined their corresponding proteins by western blot assay, as shown in Figure 5A. Compared with the sham group, the relative expression levels of AMPKβ1, p-AMPKβ1, PGC-1α and NRF2 were significantly increased, whereas the TFAM and UCP2 were attenuated in the model group, the reason for this could be the effect of receiving other signaling pathways. The relative protein expression levels of AMPKβ1, p-AMPKβ1, PGC-1α, NRF2, TFAM and UCP2 proteins were significantly increased in the rat brain tissues of the acupuncture group compared to the model group. The above results showed that mitochondrial synthesis was enhanced in MCAO rats after successful modeling, and acupuncture treatment further stimulated the expression of related proteins.

### 3.6 Acupuncture increased the expression of AMPK/PGC-1α pathway related factor RNA

To further determine the effect of acupuncture intervention on the associated neurons expression of the AMPK/PGC-1α pathway in MCAO rats, we performed quantitative Rq-PCR for AMPKβ1, PGC-1α, NRF2, TFAM, and UCP2. The results are shown in Figure 5B. I/R induced a increase in expression of AMPKβ1, PGC-1α, NRF2, TFAM, and UCP2 compared to the sham group. However, the treatment of acupuncture further stimulated the expression of AMPKβ1 and mitochondrial synthesis-related transcription factors including the PGC-1α, NRF2, TFAM and UCP2.

### 3.7 Effects of AMPK agonists and antagonists on acupuncture treatment

**Figure 6:**
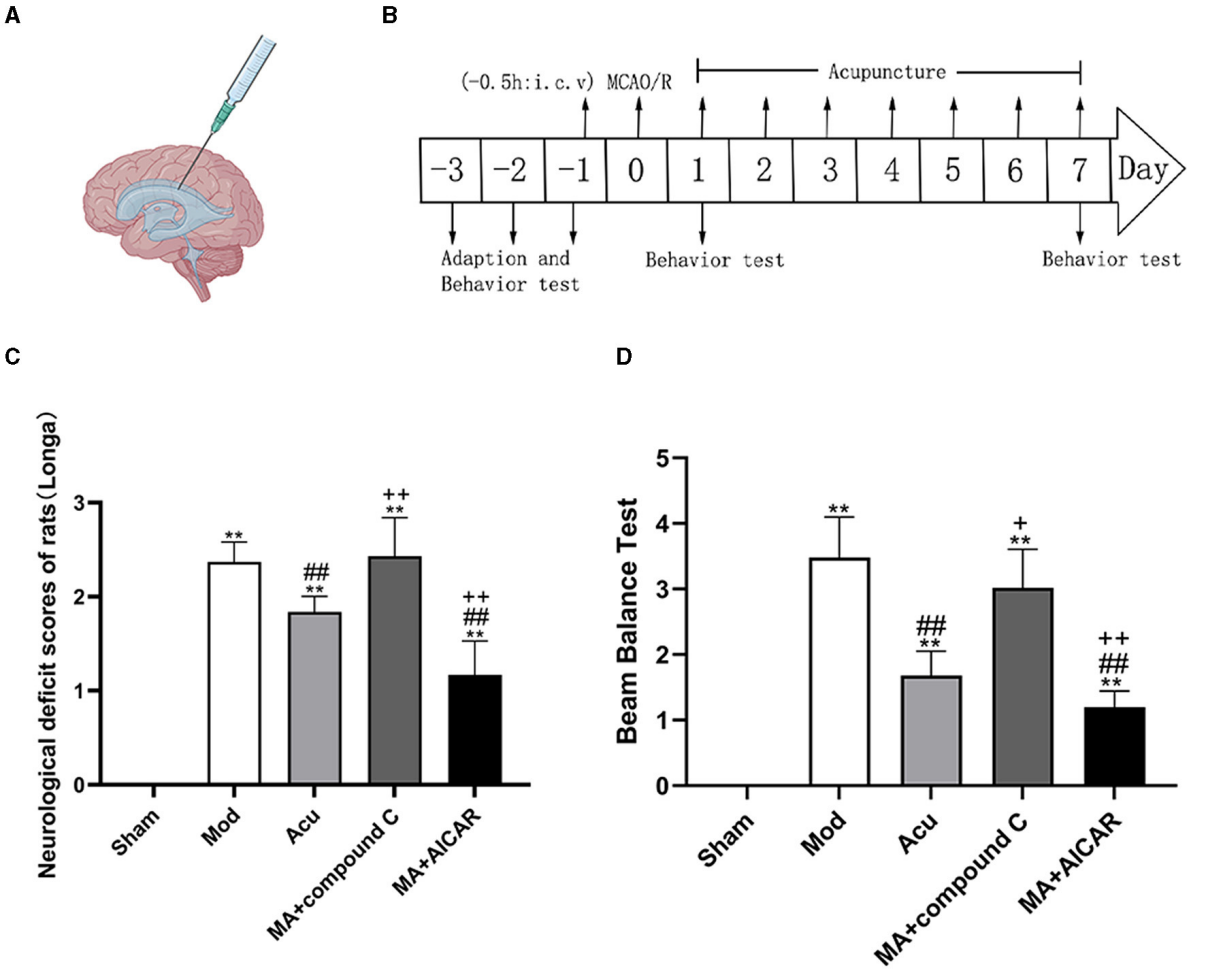
AMPK mediated the effect of acupuncture to improve neurological function injury in MCAO rodents. **(A, B)** Experimental representation of the lateral intracerebroventricular (i.c.v) and schematic representation of the experimental timeline. **(C, D)** Neurological scores of rats from each group after completion of treatment, **(C)** Longa, **(D)** Beam Balance Test (*n* = 6, one-way ANOVA). All data are presented as the means ± SEM; **P < 0.01 vs. Sham; ^##^*P* < 0.01 vs. Mod; ^+^*P* < 0.05, ^++^*P* < 0.01 vs. Acu. Compound C, an AMPK antagonist; AICAR, an AMPK agonist; MA, MCAO + Acu.

To determine whether the inhibition and activation of AMPK could affect the modulation of nerve injury by acupuncture, we observed the level of nerve injury in rats after the application of AMPK antagonists and agonists (Figure 6). Compared with the model group, rats had decreased Longa score in the acupuncture group, with increased Longa score and decreased rats in the lateral ventricle antagonists' group relative to the acupuncture group. It shows that the application of AMPK antagonist offsets the nerve injury alleviated by acupuncture, and application of AMPK agonists promote the recovery of nerve injury, thus demonstrating that AMPK may be the key target for acupuncture to ameliorate nerve injury in MCAO rats.

## 4 Discussion

Currently, there have been a large number of modern medical studies reporting on the ability of acupuncture to promote neurological recovery after stroke. For example, our previous experimental and clinical studies have confirmed that acupuncture can effectively improve various central nervous system injuries, including ischemic stroke, carbon monoxide poisoning, and Parkinson's disease (Lu et al., 2019; Tamtaji et al., 2019; Zhang et al., 2019). Many experimental studies have shown that acupuncture is effective in ameliorating neurological deficits caused by brain injury in stroke by improving cerebral blood flow and tissue oxygen supply, repairing the blood-brain barrier, improving inflammatory responses, and regulating local ion balance, among other mechanisms (Lu et al., 2019). However, there are few experimental and clinical studies on the effects of the AMPK/PGC-1α pathway in mediating mitochondrial energy metabolism on the recovery of neurological deficits after stroke. Therefore, in the present study, we focused on investigating the underlying molecular mechanisms of mitochondrial energy metabolism in the improvement of post-stroke neurological deficits by acupuncture. It was found that acupuncture treatment could improve neurological impairment in MCAO rats. To gain further insight, we adopted the fluorescence quantitative PCR, immunohistochemistry method and other methods to detect that the expression levels of factors AMPK, PGC-1α, NRF2, TFAM, and UCP2 related to the AMPK/PGC-1α pathway were all increased after acupuncture. More interestingly, the effect of acupuncture would be inhibited by AMPK antagonists. This indicates that acupuncture can activate AMPK/PGC-1 α pathways to promote neurological recovery after ischemic stroke, with AMPK as its key therapeutic target.

A systematic review and meta-analysis showed that acupuncture treatment has potential advantages in improving neuromotor function and quality of life in stroke patients (Lu et al., 2016). Multiple animal studies showed that Baihui and Zusanli acupoint can promote recovery from neurological damage in MCAO model rats by increasing the level of energy metabolism in the brain (Chen et al., 2016; Yang et al., 2021). Another study using Fengfu and Quchi acupoint found that after acupuncture intervention, the level of mitochondrial metabolism within the ischemic brain cortex increased, along with a significant improvement in neurological function in rats tested using the Longa score and balance beam test (Lu et al., 2019; Song and Ni, 2020). In the present study, we found that after acupuncture treatment for one-week, neurological function was improved in MCAO rats. Meanwhile, the results of HE staining showed that the cell damage of injured neurons in the brain of MCAO rats was significantly improved.

After ischemic stroke, the brain experiences interruption or reduction of blood flow and oxygen, insufficient supply of ATP, overproduction of reactive oxygen species (ROS), resulting in accumulation of lactate after energy deprivation, and increased levels of oxidative stress in the brain, causing the activation of NOX and other signals. NOX is the main enzyme generating ROS in addition to mitochondria in cells, and oxidative stress caused by NOX mediated ROS is increasingly recognized. Emerging evidence suggests that inhibition of elevated levels of oxidative stress may play a protective role in the treatment of complications such as stroke induced neurological injury (Kleinschnitz et al., 2010). However mitochondrial complex I is the largest multi subunit complex of the respiratory chain and one of the main motors of ATP synthesis, so its levels are positively correlated with ATP. Therefore, we adopted high-performance liquid chromatography to examine mitochondrial function, including mitochondrial respiratory chain complex I activity, intracellular NOX and ATP levels in MCAO rats. Due to the highly active level of metabolism in the brain requires a continuous supply of oxygen and energy. When oxygen and glucose reserves in the brain are insufficient, it is vulnerable to the sudden disruption of cerebral blood flow, which triggers the production of large amounts of ROS, large amounts of ROS attack the mitochondrial membrane, leading to the disruption of membrane permeability and integrity and the opening of the mitochondrial permeability transition pore (Kleinschnitz et al., 2010; Orellana-Urzúa et al., 2020), releasing a large number of proapoptotic factors (Ma et al., 2020), which together induce apoptosis through the apoptotic receptor pathway and the mitochondrial apoptotic pathway. Acupuncture not only elevates the expression levels of ATP and mitochondrial complex I, but also exerts neuroprotective functions by reducing the expression of NOX, thereby inhibiting the levels of NOS and superoxide, protecting the integrity of mitochondria. The results showed that acupuncture intervention could obviously improve mitochondrial function and enhance antioxidant capacity.

The physio pathological mechanisms of ischemic stroke involve energy metabolism and neuroinflammatory responses, in which energy metabolism plays a vital role (Domínguez et al., 2010; Sanderson et al., 2013). Compelling evidence suggests that oxidative stress involving AMPK plays a pivotal role in the brain Imbalance of energy homeostasis in the brain after cerebral ischemia, the generation and utilization of ATP are highly related to the maintenance of energy homeostasis. After cerebral ischemia, an increase in AMP/ATP or ADP/ATP ratio can activate AMPK, and the increase in AMP is always much greater than the increase in ADP or the decrease in ATP, therefore, AMPK can act as an early sensor of energy deprivation and plays a key role in cerebral energy homeostasis. Under hypoxic-ischemic conditions, AMPK plays a key role in controlling the energy axis of mitochondrial glycolysis to enhance mitochondrial biogenesis and improve intramitochondrial glucose metabolism. PGC-1α is a gatekeeper for regulating mitochondrial function, and AMPK directly affects PGC-1α activity through deacetylation and phosphorylation, which is an important link in the homeostatic regulatory network of energy metabolism. Activation of AMPK maintains the level of PGC-1 α, thus, it can enhance the mitochondrial function of cerebral ischemia, increase the energy supply, up regulate the expression of antioxidant enzymes, eliminate free radicals, improve the antioxidant stress ability of rats, reduce the degeneration and death of nerve cells, and improve the cognitive function of rats (Ham and Raju, 2017). PGC-1α is expressed in the cerebral cortex, hippocampus and cerebellum, and is neuroprotective in transient ischemic pretreated rats, associated with upregulation of PGC-1α in the hippocampus (Chen et al., 2010). In a model of permanent middle cerebral artery occlusion, enhancing the transcriptional activity of PGC-1α in neurons can improve mitochondrial dysfunction and oxidative damage and exert neuroprotective effects (Fu et al., 2014). PGC-1a can gradually activate the promoters of NRF2 and TFAM, produce NRF2 and TFAM protein factors, transport and bind mitochondrial DNA, and finally promote the transcription of mitochondria (Zhang and Liang, 2019). UCP, an uncoupling protein located on the inner mitochondrial membrane, protects mitochondria in situations of oxidative stress. Phosphorylated AMPK and PGC-1 act against oxidative stress and promote mitochondrial neogenesis by increasing UCP2, mitochondrial respiratory chain complex I expression and eliminating ROS (Besseiche et al., 2018).

Phosphorylation-AMPK and its downstream proteins act as a powerful metabolic regulator that is widely involved in mitochondrial energy metabolism and plays a key role in the recovery of injured neurons (Young et al., 2016). Subsequently, after modeling in addition to TFAM, AMPK, PGC-1 α and increased secretion stress of each transcription factor within mitochondria. After acupuncture intervention, AMPK phosphorylation was enhanced, and the secretion of PGC-1α, UCP2, NRF2, and TFAM proteins was significantly increased. Thus, our results suggest that it can enhanced release of mitochondrial transcription factors, promote mitochondrial biogenesis and energy metabolism in the brain. In the injured cerebral cortical area, an adequate energy supply favors the recovery and reconstruction of the injured neurons.

After discovering that acupuncture can promote AMPK phosphorylation and a significant increase in AMPK/PGC-1α pathway related mitochondrial transcription factors, combined with previous PCR analysis results, we hypothesized that AMPK is a key target. Subsequently, we injected the AMPK antagonist and agonists through the lateral ventricle into acupuncture-treated MCAO rats to confirm the mediating effect of this factor. The results showed that the AMPK antagonist effectively blocked the acupuncture effect, AMPK agonists enhance the acupuncture effect, indicating that AMPK mediates the recovery of nerve injury from acupuncture treatment and is a key target for the treatment of ischemic stroke. Activation of AMPK can promote autophagy to compensate for energy demand in cells, and acupuncture can inhibit excessive autophagy in cranial neurons by regulating AMPK activation, thereby exerting a neuroprotective effect (Huang et al., 2019; Gu et al., 2020).

## 5 Conclusion

**Figure 7:**
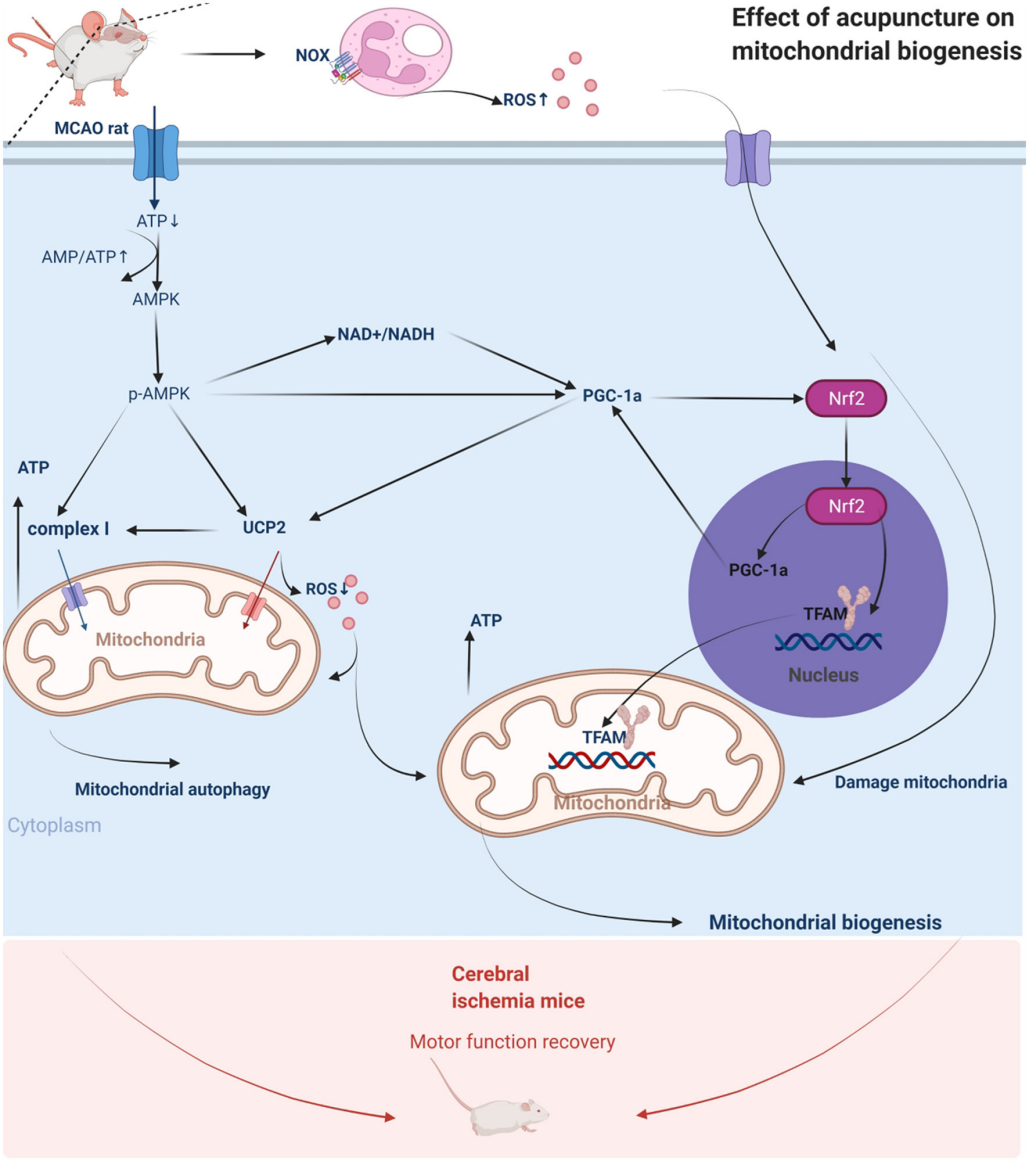
Acupuncture intervention promotes mitochondrial biogenesis mechanism.

Given our previous findings that acupuncture ameliorates symptoms of neurological damage in patients with ischemic stroke, our present work elucidates that acupuncture at Fengfu acupoints (GV16), Baihui acupoints (GV20) and acupoints including bilateral Fengchi (GB20), Zusanli (ST36), and Quchi acupoints (LI11). The present study suggests that acupuncture can directly activate AMPK and activate downstream PGC-1α and mitochondrial transcription related factors or inhibit the body's NOx levels and thereby reduce the excessive accumulation of ROS after ischemia to activate the AMPK/PGC-1α axis. Ultimately, it promotes the release and biosynthesis of downstream mitochondrial transcription factors, increases the generation of ATP, inhibits the excessive accumulation of ROS, promotes mitochondrial energy metabolism and biogenesis, maintains cellular homeostasis, inhibits neuronal apoptosis, improves brain energy metabolism, and promotes recovery from neurological injury. The regulatory mechanism of acupuncture on mitochondria in ischemic neurons is shown in Figure 7. Furthermore, our findings also show that, AMPK plays a key therapeutic target role in the cerebral cortex. These findings provide an innovative scientific basis for the clinical application of acupuncture to promote recovery from neurological damage after stroke.

## Data availability statement

The original contributions presented in the study are included in the article/Supplementary material, further inquiries can be directed to the corresponding author.

## Ethics statement

The animal study was reviewed and approved by experimental animal welfare Ethics Review Committee, Shandong University of Traditional Chinese Medicine (SDUTCM20220302042). The study was conducted in accordance with the local legislation and institutional requirements.

## Author contributions

KG: Data curation, Investigation, Project administration, Software, Validation, Writing—original draft, Writing—review & editing. YL: Conceptualization, Formal analysis, Funding acquisition, Methodology, Resources, Supervision, Visualization, Writing—original draft, Writing—review & editing.
