# Supplementary material for: Acupuncture modulates the AMPK/PGC-1 signaling pathway to facilitate mitochondrial biogenesis and neural recovery in ischemic stroke rats
Source: Front Mol Neurosci. 2024 May 15;17:1388759. doi: 10.3389/fnmol.2024.1388759 (PMC11133568; doi:10.3389/fnmol.2024.1388759)
Supplement: Supplementary file 1 [file Table_1.docx]

Supplementary table1 The primer sequences

| Primer information | Primer names | Primer sequence（5'-3') | Length of fragment（bp） | Temperature of annealing（℃） |
| --- | --- | --- | --- | --- |
| [NM_031976.2](https://www.ncbi.nlm.nih.gov/nuccore/NM_031976.2) | R-Prkab1-S | CGAGCCAATAGTAACCAGCCA | 99 | 60 |
| AMPKb1 | R-Prkab1-A | TGGGAATCCACCATTAAAGCA |  | 60 |
| [NM_031347.1](https://www.ncbi.nlm.nih.gov/entrez/viewer.fcgi?db=nucleotide&id=13786187) | R-PGC1a(1)-S | GAGAAGCGGGAGTCTGAAAGG | 219 | 60 |
|  | R-PGC1a(1)-A | GTCACAGGTGTAACGGTAGGTAATG |  | 60 |
| [NM_031789.2](https://www.ncbi.nlm.nih.gov/entrez/viewer.fcgi?db=nucleotide&id=402692377) | R-nrf2(6)-S | AATTGCCACCGCCAGGACT | 100 | 60 |
|  | R-nrf2(6)-A | TCAAACACTTCTCGACTTACCCC |  | 60 |
| [NM_031326.2](https://www.ncbi.nlm.nih.gov/nuccore/NM_031326.2) | R-TFAM-S | GGCGTGCTAAGAACACTGGG | 87 | 60 |
|  | R-TFAM-A | ACAGATAAGGCTGACAGGCGAG |  | 60 |
| NM_019354.3 | R-UCP2-S | TCCCAATGTTGCCCGAAATG | 99 | 60 |
|  | R-UCP2-A | TCGTCTGTCATGAGGTTGGC |  | 60 |
| NM_017008.4 | R-GAPDH-S | CTGGAGAAACCTGCCAAGTATG | 138 | 60 |
|  | R-GAPDH-A | GGTGGAAGAATGGGAGTTGCT |  | 60 |
